# Supplementary material for: Competing health risks associated with the COVID-19 pandemic and early response: A scoping review
Source: PLoS One. 2022 Aug 29;17(8):e0273389. doi: 10.1371/journal.pone.0273389 (PMC9423636; doi:10.1371/journal.pone.0273389)
Supplement: S1 Appendix — (DOCX) [file pone.0273389.s003.docx]

**APPENDIX: Search Strategies**

**HIV**

((("COVID-19"[tw] OR "COVID 19"[tw] OR "COVID19"[tw] OR "COVID2019"[tw] OR "COVID 2019"[tw] OR "COVID-2019"[tw] OR "novel coronavirus"[tw] OR "new coronavirus"[tw] OR "novel corona virus"[tw] OR "new corona virus"[tw] OR "SARS-CoV-2"[tw] OR "SARSCoV2"[tw] OR "SARS-CoV2"[tw] OR "2019nCoV"[tw] OR "2019-nCoV"[tw] OR "2019 coronavirus"[tw] OR "2019 corona virus"[tw] OR "coronavirus disease 2019"[tw] OR "severe acute respiratory syndrome coronavirus 2"[nm] OR "severe acute respiratory syndrome coronavirus 2"[tw] OR "sars-coronavirus-2"[tw] OR "coronavirus disease 2019"[tw] OR "corona virus disease 2019"[tw])) AND (("2020/MM/DD"[PDAT] : "3000/MM/DD"[PDAT]))) AND ("HIV"[Mesh] OR "Acquired Immunodeficiency Syndrome"[Mesh] OR "HIV Infections"[Mesh] OR human immunodeficiency virus*[tw] OR acquired immunodeficiency syndrome*[tw] OR HIV*[tw] OR "AIDS"[tw] OR HIV1*[tw] OR HIV2*[tw])

**Malnutrition**

((("COVID-19"[tw] OR "COVID 19"[tw] OR "COVID19"[tw] OR "COVID2019"[tw] OR "COVID 2019"[tw] OR "COVID-2019"[tw] OR "novel coronavirus"[tw] OR "new coronavirus"[tw] OR "novel corona virus"[tw] OR "new corona virus"[tw] OR "SARS-CoV-2"[tw] OR "SARSCoV2"[tw] OR "SARS-CoV2"[tw] OR "2019nCoV"[tw] OR "2019-nCoV"[tw] OR "2019 coronavirus"[tw] OR "2019 corona virus"[tw] OR "coronavirus disease 2019"[tw] OR "severe acute respiratory syndrome coronavirus 2"[nm] OR "severe acute respiratory syndrome coronavirus 2"[tw] OR "sars-coronavirus-2"[tw] OR "coronavirus disease 2019"[tw] OR "corona virus disease 2019"[tw])) AND (("2020/MM/DD"[PDAT] : "3000/MM/DD"[PDAT]))) AND ("Infant nutrition disorders"[Mesh] OR "child nutrition disorders"[Mesh] OR "malnutrition"[Mesh] OR "thinness"[Mesh] OR "wasting syndrome" [Mesh] OR "undernutrition"[tw] OR undernourish*[tw] OR "chronic energy deficiency"[tw] OR "stunting"[tw] OR "wasting"[tw] OR "underweight"[tw] OR "thinness"[tw] OR micronutrient deficienc*[tw] OR vitamin deficienc*[tw] OR mineral deficienc*[tw])

**Malaria**

((("COVID-19"[tw] OR "COVID 19"[tw] OR "COVID19"[tw] OR "COVID2019"[tw] OR "COVID 2019"[tw] OR "COVID-2019"[tw] OR "novel coronavirus"[tw] OR "new coronavirus"[tw] OR "novel corona virus"[tw] OR "new corona virus"[tw] OR "SARS-CoV-2"[tw] OR "SARSCoV2"[tw] OR "SARS-CoV2"[tw] OR "2019nCoV"[tw] OR "2019-nCoV"[tw] OR "2019 coronavirus"[tw] OR "2019 corona virus"[tw] OR "coronavirus disease 2019"[tw] OR "severe acute respiratory syndrome coronavirus 2"[nm] OR "severe acute respiratory syndrome coronavirus 2"[tw] OR "sars-coronavirus-2"[tw] OR "coronavirus disease 2019"[tw] OR "corona virus disease 2019"[tw])) AND (("2020/MM/DD"[PDAT] : "3000/MM/DD"[PDAT]))) AND ("Malaria" [Mesh] OR "malaria" [tw] OR "plasmodium" [tw])

**TB**

((("COVID-19"[tw] OR "COVID 19"[tw] OR "COVID19"[tw] OR "COVID2019"[tw] OR "COVID 2019"[tw] OR "COVID-2019"[tw] OR "novel coronavirus"[tw] OR "new coronavirus"[tw] OR "novel corona virus"[tw] OR "new corona virus"[tw] OR "SARS-CoV-2"[tw] OR "SARSCoV2"[tw] OR "SARS-CoV2"[tw] OR "2019nCoV"[tw] OR "2019-nCoV"[tw] OR "2019 coronavirus"[tw] OR "2019 corona virus"[tw] OR "coronavirus disease 2019"[tw] OR "severe acute respiratory syndrome coronavirus 2"[nm] OR "severe acute respiratory syndrome coronavirus 2"[tw] OR "sars-coronavirus-2"[tw] OR "coronavirus disease 2019"[tw] OR "corona virus disease 2019"[tw])) AND (("2020/MM/DD"[PDAT] : "3000/MM/DD"[PDAT]))) AND ("Tuberculosis" [Mesh] OR "tuberculosis" [tw] OR "TB"[tw] OR mycobacter*[tw])

Sexual and Reproductive Health

((("COVID-19"[tw] OR "COVID 19"[tw] OR "COVID19"[tw] OR "COVID2019"[tw] OR "COVID 2019"[tw] OR "COVID-2019"[tw] OR "novel coronavirus"[tw] OR "new coronavirus"[tw] OR "novel corona virus"[tw] OR "new corona virus"[tw] OR "SARS-CoV-2"[tw] OR "SARSCoV2"[tw] OR "SARS-CoV2"[tw] OR "2019nCoV"[tw] OR "2019-nCoV"[tw] OR "2019 coronavirus"[tw] OR "2019 corona virus"[tw] OR "coronavirus disease 2019"[tw] OR "severe acute respiratory syndrome coronavirus 2"[nm] OR "severe acute respiratory syndrome coronavirus 2"[tw] OR "sars-coronavirus-2"[tw] OR "coronavirus disease 2019"[tw] OR "corona virus disease 2019"[tw])) AND (("2020/MM/DD"[PDAT] : "3000/MM/DD"[PDAT]))) AND ("Reproductive Health" [Mesh] OR "Reproductive Health Services"[Mesh] OR “maternal health”[Mesh] OR “maternal health services”[Mesh] OR "reproductive health"[tw] “maternal health”[tw] OR contracept*[tw] OR "family planning"[tw] OR "abortion"[tw] OR "post-abortion care"[tw] OR "unintended pregnancy"[tw] OR “unplanned pregnancy”[tw] OR “unwanted pregnancy”[tw])
