# Supplementary material for: Competing health risks associated with the COVID-19 pandemic and early response: A scoping review
Source: PLoS One. 2022 Aug 29;17(8):e0273389. doi: 10.1371/journal.pone.0273389 (PMC9423636; doi:10.1371/journal.pone.0273389)
Supplement: Attachment — Submitted filename: Response to Reviewers.docx [file pone.0273389.s004.docx]

PONE-D-21-08875 (Revision due Dec 05 2021, 11:59 PM)

Competing Health Risks Associated with the COVID-19 Pandemic and Response: A Scoping Review

We thank the reviewer for taking the time to provide a careful review of our paper. We have responded in detail, point-by-point below.

Reviewer #1: 1. Line 133 and 134 do not state clearly the period that the authors concentrated in their search for published literature and preprints. Just stating “through October 31st” is not enough and is, in fact, ambiguous

Thank you for this point, and we agree that the way this is stated is ambiguous. We have revised this to define the search period more clearly (now lines 144-145).

2. In lines 182 and 183, the authors make a statement about the decrease in dispensing of pre-exposure prophylaxis claiming it might be due to reduced sexual activity, this statement should probably be referenced or removed entirely with the authors sticking to the referenced materials since this is not the discussion section of the paper

Thank you for this point. We had noted this point about a decrease in sexual activity, in conjunction with the decline in PrEP results, as this was also a primary result observed and presented in the papers reporting declines in PrEP and PEP use. We have now more clearly referenced this statement and added an additional example to clarify the point:

*“For example, one study of 847 gay and bisexual men in Australia found that about 42% discontinued PrEP use during the COVID-19 pandemic and that those discontinuing were less likely to report casual sexual partners [23].”*

3. It will also be good if the use some actual values from the quoted sources to show the decrease number of persons being tested and also accessing pre-exposure prophylaxis

Thank you for this suggestion. We have now provided some specific values on the decrease in testing and PrEP use seen during the COVID-19 pandemic.

4. Concerning lines 225 and 226, which period of the year are the authors referring to? Do they mean the whole of 2020? Nigeria, for instance, had an overall increase in TB case finding in 2020 compared to previous years. The authors should look at the statement and consider rephrasing it to reflect the period in 2020 they were referring to.

Thank you for this suggestion. We have revised this statement to clearly indicate which period of 2020 we are referring to here. The statement now reads: *“For instance, in early 2020, there had been a substantial reduction in TB notifications in Sierra Leone, China, Nigeria, South Korea, India, the Philippines, Japan, and the U.S. compare do the same period in previous years [48-57].”*

5. The opening statements of the discussion seems like something that would have fitted well as part of the background for this study. It sounds like a more catchy way of introducing this study.

We agree with your assessment and have changed the opening lines of the discussion to focus on summarizing the preliminary and overarching findings of the Review.

**ADDITIONAL EDITORIAL COMMENTS**

We note that the grant information you provided in the ‘Funding Information’ and ‘Financial Disclosure’ sections do not match.

When you resubmit, please ensure that you provide the correct grant numbers for the awards you received for your study in the ‘Funding information section’.

We have updated the funding information sections and financial disclosure sections to reflect the most recent information.

Please complete your Competing Interests on the online form to state any Competing Interests. If you have no competing interests, please state "The authors have declared that no competing interests exist.", as detailed online in our guide for authors at [http://journals.plos.org/plosone/s/submit-now](https://nam02.safelinks.protection.outlook.com/?url=http%3A%2F%2Fjournals.plos.org%2Fplosone%2Fs%2Fsubmit-now&data=04%7C01%7Carao24%40jhu.edu%7Cea2e09e00e5e44a6074a08d9a7be59ca%7C9fa4f438b1e6473b803f86f8aedf0dec%7C0%7C0%7C637725258293567515%7CUnknown%7CTWFpbGZsb3d8eyJWIjoiMC4wLjAwMDAiLCJQIjoiV2luMzIiLCJBTiI6Ik1haWwiLCJXVCI6Mn0%3D%7C3000&sdata=kbOL6yBeNLqv53uXt8G3IuyQBlAYqs%2FrzObd%2B56bYvg%3D&reserved=0) 
 This information should be included in your cover letter; we will change the online submission form on your behalf.

Thank you for this. We have provided updated competing interests in our resubmission cover letter.

In your Data Availability statement, you have not specified where the minimal data set underlying the results described in your manuscript can be found. PLOS defines a study's minimal data set as the underlying data used to reach the conclusions drawn in the manuscript and any additional data required to replicate the reported study findings in their entirety. All PLOS journals require that the minimal data set be made fully available. For more information about our data policy, please see [http://journals.plos.org/plosone/s/data-availability](https://nam02.safelinks.protection.outlook.com/?url=http%3A%2F%2Fjournals.plos.org%2Fplosone%2Fs%2Fdata-availability&data=04%7C01%7Carao24%40jhu.edu%7Cea2e09e00e5e44a6074a08d9a7be59ca%7C9fa4f438b1e6473b803f86f8aedf0dec%7C0%7C0%7C637725258293567515%7CUnknown%7CTWFpbGZsb3d8eyJWIjoiMC4wLjAwMDAiLCJQIjoiV2luMzIiLCJBTiI6Ik1haWwiLCJXVCI6Mn0%3D%7C3000&sdata=YsxtAXZO%2Bi%2FzBbLoJjWUcX4wph5Gw07vU1ZWLhrQZRY%3D&reserved=0).

We have now provided a complete list of all the papers included in our scoping review in a Supporting Information File and updated the Data Availability statement.

Please include captions for your Supporting Information files at the end of your manuscript, and update any in-text citations to match accordingly. Please see our Supporting Information guidelines for more information: [http://journals.plos.org/plosone/s/supporting-information](https://nam02.safelinks.protection.outlook.com/?url=http%3A%2F%2Fjournals.plos.org%2Fplosone%2Fs%2Fsupporting-information&data=04%7C01%7Carao24%40jhu.edu%7Cea2e09e00e5e44a6074a08d9a7be59ca%7C9fa4f438b1e6473b803f86f8aedf0dec%7C0%7C0%7C637725258293577473%7CUnknown%7CTWFpbGZsb3d8eyJWIjoiMC4wLjAwMDAiLCJQIjoiV2luMzIiLCJBTiI6Ik1haWwiLCJXVCI6Mn0%3D%7C3000&sdata=3%2BQCLNUNXYJrvgU%2F0D5jcKSsTP8yCnAf2X7uOxbOTMU%3D&reserved=0).

Thank you for this. We have added a caption for the appendix file that provides the search strategies used for this scoping review.

We note the search of the relevant literature is limited to searches on PubMed. We do not feel that this constitutes a search that is comprehensive enough to collect all relevant evidence available from the literature, and as a result, we have concerns about the strength of the conclusions drawn from the pool of studies included in your synthesis. Therefore, please extend your search to include other relevant databases and include any additional papers in your review, if applicable.

Thank you for this feedback. Our scoping review includes papers identified through a systematic search of PubMed, along with a comprehensive search of the COVID-19 Living Evidence Database, which includes published papers and preprints from both medRxiv and bioRxiv and is updated daily. We do agree with your point that some papers not indexed in PubMed may not have been included in our review, and so we have added a note in the Limitations section detailing this.

In your methods section, please report the inclusion and exclusion criteria used in your review.

Thank you for this. We have included the inclusion and exclusion criteria for this review in lines 140-145 of the Methods section. We have also provided the text below:

*Articles were eligible for inclusion if the primary focus was the impact of COVID-19 on one of the five listed public health threats, and if they included empiric data or mathematical models on health or service outcomes. We excluded articles not written in English. Finally, commentaries were screened for key themes to support interpretation of the measured and modeled data. The most recent search was executed on October 31, 2020.*
