# Supplementary material for: Competing health risks associated with the COVID-19 pandemic and early response: A scoping review
Source: PLoS One. 2022 Aug 29;17(8):e0273389. doi: 10.1371/journal.pone.0273389 (PMC9423636; doi:10.1371/journal.pone.0273389)
Supplement: Attachment — Submitted filename: Response to reviewers_30June2022.docx [file pone.0273389.s005.docx]

**PONE-D-21-08875R1**

**Competing Health Risks Associated with the COVID-19 Pandemic and Early Response: A Scoping Review**

Due July 18, 2022

- Response to reviewers
- Revised Manuscript with Track Changes
- Manuscript (Clean)

**REVIEW COMMENTS TO THE AUTHOR**

**Reviewer #1**: This paper has been properly written. All the previous concerns i had, especially in the presentation of the results were addressed by the authors.

**Reviewer #2**:

**Abstract**
-It is very confusing the figures got from their literature search when mentioned. 1604 published papers and 205 preprints met inclusion criteria, but only 132 and 21 were selected, respectively. They have to replace the verb “including” by “selecting”.

Thank you for this comment. We included articles that had a primary focus on the impact of COVID-19 on one of the five listed public health threats AND if they included empiric data or mathematical models on health or service outcomes. We realize there is some confusion with the phrase “met our inclusion criteria,” and have revised this section of the abstract to the following:

*“A total of 1604 published papers and 205 preprints were* ***retrieved in the search****. Overall, 8.0% (129/1604) of published studies and 10.2% (21/205) of preprints* ***met the inclusion criteria and were included in this review****…”*

**Introduction**
-References are not properly used in some instances. Reference [2] is not related to Public Health responses but to estimating the success of HIV care in key population.

Thank you for catching this. There was an error that occurred when transferring our references between versions. We have now corrected this to include the correct reference:

Hale, T, Angrist, N, Goldszmidt, R, Kira, B, Petherick, A, Phillips, T, et al. A global panel database of pandemic policies (Oxford COVID-19 Government Response Tracker). Nat Hum Behav. doi: 10.1038/s41562-021-01079-8

-Lines 123-124. Delete “rather than systematic review”. If they want to discuss this point, relocate it in Discussion section.

Per your suggestion, we removed “rather than systematic review” from this sentence. We agree that mention of this should be restricted to the Discussion section, which is included in the Limitation sub-section.

-**Methods**
-Line 130. The other reviewer has already pointed that this sentence is confusing or trifling. In my opinion, they should merge the first sentence and the following one, or simply delete the first one.

We have revised this sentence to merge the first and the following sentence.

-Line 131. They must enter the MeSH acronym the first time it was used in the text, as they did it in Abstract.

Thank you for this. We have added what MeSH stands for here.

-There is a contradictory information regarding period of inclusion. In Abstract, the period cited ranged from January 1st to October 31st, 2020, whereas in Methods (line 144) the period started two months earlier (November 2019).

Thank you for catching this. This should read November 2019 throughout, and we have corrected this.

-Paragraph from line 140 to 145. They must merge inclusion criteria and then, cite the exclusion criterion (this appears in the middle of the paragraph now, line 142).

Thank you for this suggestion. We moved the last sentence indicating the timeline for the review up to be immediately after the other inclusion criteria. The paragraph now reads as follows:

Articles were eligible for inclusion if the primary focus was the impact of COVID-19 on one of the five listed public health threats, and if they included empiric data or mathematical models on health or service outcomes. Articles were included if they were published or posted between November 2019 and October 2020 with the most recent search implemented on October 31, 2020. We excluded articles not written in English. Commentaries were screened for key themes to support interpretation of the measured and modeled data.

**Results**
-I have missed a summary table or a general summary of all the findings.

We have presented a summary of findings at the beginning of each health risk section. We did indicate that “COVID-19 has been associated with reduced access to services, decreased diagnosis, and poorer health outcomes for HIV, TB, malaria, and SRH, and increases in malnutrition.”

We did not include any further summary of the findings across health risks, given concerns about the oversimplification of the results and earlier feedback about repetition across sections of the manuscript.

-There is a huge difference between the number of articles retrieved and that finally selected, so another exclusion criteria besides non-English language may have been used. They should specify why they discarded so many articles.

Thank you for this comment. We noted in the Methods section that the inclusion criteria also stipulated that articles include empiric data or mathematical modeling data. This was the primary factor for determining whether or not articles were included in our review. This requirement has now been clarified, as noted above.

-Line 162. According to Table 1, the figures given for articles retrieved and selected for SRH is not correct (13/166). Table 1 shows 19 papers included out of a total of 183.

Thank you very much for catching this error; we also subsequently noted this error in the abstract and have revised it in both places.

-The way of citation along the text is not consistent. For instance, the bracket including references 13 and 14 must be placed before the full stop (line 172). The same for reference [18] in line 178, [16] in line 186, [14] and [27] in line 189, etc. Citations are sometimes after, sometimes before full stops.

Thank you for this. This manuscript was the result of the collaborative work of all of the authors using different styles; we have gone through and corrected the inconsistencies.

-Line 177. 820 out of 1254 is more than a half, so they must write it in that way [more than a half reported being unable…].

We have revised this to specify “more than half…” rather than just “half.”

-Line 190. People who use drugs must be specified that these are injected (intravenous drug users).

Here, we use the phrase “people who use drugs” to include both those who inject drugs and those who do not. Lack of access to services during this period was particularly problematic for those who already had a difficult time engaging in services in the first place, and this included people who used drugs more broadly.

-Lines 205-206. They must specify the key population of this study.

The study referenced here does not refer to a specific key population, but rather to a population of individuals living with HIV being served by a safety-net HIV clinic in San Francisco. It includes those that are receiving publicly funded insurance and have a higher prevalence of mental illness, substance use, and unstable housing.

-Line 213. The WHO goal to finish the TB pandemic must be referenced with the corresponding citation.

We have revised this sentence and included a citation as appropriate.

-lines 241 and 242. The sentence referring to losses of jobs should be related to TB. Otherwise, it is not consistent there.

Good point. We have revised this sentence to relate job loss to risk of TB.

-Lines 324 and 325. They must replace each citation after a country with the citation at the end of the sentence, as they already did before in the previous paragraphs.

We have moved these citations so that they are within the comma for each country, both for consistency across the paper and to clarify any confusion.

-Line 130. I cannot find the relationship between reference 130 and the impact of school closures in children´s nutrition. Is this reference correctly used?

Thank you. Upon reviewing this further, we realized that this citation (130) was meant to be used in a previous line, indicating that food insecurity could be related to disruption of food supply chains because of limited movement of people and goods and disruption of food markets. We have made this correction.

-line 357. Replace “less than five years” by “younger than five years”.

Thank you; we have made this change.

-Lines 359 to 364. They have to insert a citation related to this statement, before writing about maternal mortality.

Thank you very much for this careful review of the references. We have added the appropriate citations to this section.

-Lines 383 and 384. The second part of the sentence (after comma) doesn´t have any reference. Is the reference [136] also suitable? In that case, this reference must be placed at the end of the sentence.

Your interpretation is correct; we have moved the reference to the end of the sentence.

**-References**
Date of access to webpages must be included at the end of citation (i.e., [9], [138], [145], [146], [160], and so on).

We have gone through all of the citations and added access dates from the following references: 6, 8, 9, 93, 138, 145, 146, 158, 160.

[52], [62]. Pages are missing.

Pages have been added, along with volume and issue numbers.

[11]. It is not properly a reference, but a product, a software, without referring to any author. Remove this reference and take the information to the text between brackets. The same as EndNote, reference

[12].

We have consistently cited both EndNote and Covidence as references in prior review papers and feel that it is a reasonable approach for the purposes of a review such as this one. However, we defer to the editors to alert us if the journal has a preference on this point and we would be happy to adjust.

[145]. It is not exactly correct how this reference is depicted. Is it a link to a webpage? Must indicate the page and date of access.

We have added a link to the webpage where the report can be found and added an access date.

-Table 1. I am not sure of “pulled” was the most accurate term. I would rather use “retrieved”. In addition, authors must add an extra column to depict the total amount of each category (included, commentaries, excluded, etc.) and risk condition (HIV, malaria, etc), both published and preprints literature.

Thank you for this. We have changed “pulled” to “retrieved” and added a total column for each risk condition. It would not make sense to add a total column for included, commentaries, excluded as that is reflected in the number retrieved.
